# Collaborative mining of public data resources in neuroinformatics

**DOI:** 10.3389/fnins.2015.00090

**Published:** 2015-03-18

**Authors:** Rupert W. Overall, Robert W. Williams, J. Alexander Heimel

**Affiliations:** ^1^Center for Regenerative Therapies Dresden, Genomics of Regeneration, Technische Universität DresdenDresden, Germany; ^2^Department of Genetics, Genomics and Informatics, University of Tennessee Health Science CenterMemphis, TN, USA; ^3^Cortical Structure and Function Group, Netherlands Institute for NeuroscienceAmsterdam, Netherlands

**Keywords:** data analysis, online resources, transcriptomics, proteomics, brain disease, systems genetics

Modern biology is marked by the availability of enormous quantities of raw data. Advances in computer capacity and internet bandwidth mean that much of this information is now stored online, and most such data repositories are also open access. Together, these factors have opened up a goldmine of data to anyone with an internet connection. One of the greatest strengths of the available resources is their diversity; freely-accessible repositories exist, for example, for gene sequence, mRNA expression, protein binding, imaging, and text mining results. Standardized annotation schemes have also improved compatibility across different fields. The ability to obtain a wealth of information for any gene and its molecular interactions now enables a whole new realm of analysis beyond that originally envisaged by the individual contributors.

In fact, so much untapped data exists that primary research can now conceivably be carried out purely *in silico*. Because this approach to science utilizes data already available in the public domain and requires only a laptop connected to the internet, analyses are not subject to the constraints of a traditional “bricks-and-mortar” laboratory. Collaborating scientists may be located anywhere in the world, they have no need to invest in further data generation, and may be largely independent of their local funding environment. These factors thus open up access to the science to a wider research community than ever before.

Despite the wealth of public data, often accessible through user-friendly portals, there is still a “missing link” between data and discovery. Few researchers are aware of the resources or how to use them effectively. Thus, the bottleneck has shifted from the generation of raw data to the development of effective ways to explore, mine, and integrate existing information.

In September 2013, a 1-week workshop was held to introduce researchers and students to the use of such online resources (visit https://sites.google.com/site/neuroinformaticsjamboree/ for details and a list of resource links). Participants were shown how key resources can be exploited as either supporting data for existing projects or for hypothesis generation to kick-start new directions in their research (see the associated Workshop Report, Heimel et al., 2014). As an extension of this workshop, an experiment in collaborative science was undertaken, in which researchers were invited to investigate workflows with which online resources could be mined to yield new hypotheses.

The manuscripts resulting from this experiment, and presented in this Research Topic, show that online data resources in the hands of energetic investigators can yield interesting and relevant results in very short order. Drafts of these papers were all produced from scratch in 1 week and following significant work over the following year, all groups successfully converted their drafts into peer-reviewed publications. The six resulting manuscripts present a varied range of insights into the genetics of brain function and disease.

Vied and colleagues have undertaken a novel *in silico* investigation of neocortical development utilizing the Allen Institute's Developing Mouse Brain Atlas and the BrainSpan Atlas of the Developing Human Brain. Based on correlating *in situ* expression profiles, they propose a number of candidate genes with expected roles in embryonic development of the neocortex (Vied et al., 2014). Lotan and colleagues have integrated catalogs of genes associated with psychiatric disorders to search for common processes. They show that many genes associated with multiple disorders are localized to the postsynaptic density (Lotan et al., 2014). Pietrzykowski and Spijker investigated another aspect of neuropsychiatric disorders, impulsivity, by searching the GeneNetwork database for behavior and expression correlates to identify several microRNAs with putative roles in the regulation of impulse control (Pietrzykowski and Spijker, 2014). Capurro and colleagues used a novel approach to investigate genes involved in movement disorders. They employed computational deconvolution of gene expression data from the brains of Huntington and Parkinson patients to identify genes and pathways with altered expression in these disorders (Capurro et al., 2014). To explore gene regulation in autism, van de Lagemaat and colleagues studied transcriptional changes in synaptic transmission pathways. Using gene lists curated from the literature and published databases, they discovered an age-dependent imbalance in expression of genes associated with excitatory and inhibitory synapses (van de Lagemaat et al., 2014). Ashbrook and colleagues searched for novel genes associated with adult hippocampal neurogenesis. Defining a list of genes correlating with nestin, an established precursor cell marker, and highly expressed in the hippocampal dentate gyrus, they identified potential novel genes associated with the proliferating precursor cell population (Ashbrook et al., 2014).

The wide sample of web-based data sets and tools used in these studies is certainly not an exhaustive list, and many more resources exist—most of which are freely accessible. While the body of public data is dominated by large-scale initiatives, even more data are potentially available as part of smaller projects—although accessibility is still dependent on improving data-sharing standards (Ferguson et al., 2014; Sejnowski et al., 2014). We feel it is important that the studies presented in this Research Topic relied solely on existing public data. This fact demonstrates that the utility of such data sets does not need to end with initial publication—as has traditionally been the case in the past.

With six solid contributions to the literature, we feel that the experiment has been a success. We hope that this project will inspire other researchers to take advantage of the online resources available to them to inspire and complement their experimental work.

## Conflict of interest statement

The authors declare that the research was conducted in the absence of any commercial or financial relationships that could be construed as a potential conflict of interest.
